# Nano-Encapsulation of Arsenic Trioxide Enhances Efficacy against Murine Lymphoma Model while Minimizing Its Impact on Ovarian Reserve *In Vitro* and *In Vivo*

**DOI:** 10.1371/journal.pone.0058491

**Published:** 2013-03-20

**Authors:** Richard W. Ahn, Susan L. Barrett, Meera R. Raja, Jennifer K. Jozefik, Lidia Spaho, Haimei Chen, Marcel B. Bally, Andrew P. Mazar, Michael J. Avram, Jane N. Winter, Leo I. Gordon, Lonnie D. Shea, Thomas V. O’Halloran, Teresa K. Woodruff

**Affiliations:** 1 Department of Chemistry, Chemistry of Life Processes Institute, Northwestern University, Evanston, Illinois, United States of America; 2 Robert H. Lurie Comprehensive Cancer Center, Northwestern University, Chicago, Illinois, United States of America; 3 Member of the Oncofertility Consortium, Northwestern University, Chicago, Illinois, United States of America; 4 Department of Obstetrics and Gynecology, Northwestern University, Chicago, Illinois, United States of America; 5 Center for Reproductive Science Reproductive Biology Training Program, Northwestern University, Evanston, Illinois, United States of America; 6 Centre for Drug Research and Development, University of British Columbia, Vancouver, British Columbia, Canada; 7 Department of Anesthesiology and Mary Beth Donnelley Clinical Pharmacology Core Facility of the Robert H. Lurie Comprehensive Cancer Center of the Northwestern University Feinberg School of Medicine, Chicago, Illinois, United States of America; 8 Department of Medicine, Division of Hematology/Oncology and Lymphoma Program, Northwestern University Feinberg School of Medicine, Chicago, Illinois, United States of America; 9 Department of Chemical and Biological Engineering, Northwestern University, Evanston, Illinois, United States of America; 10 Department of Advanced Therapeutics, BC Cancer Agency, British Columbia, Canada; LAAS-CNRS, France

## Abstract

Advances in cancer therapy have increased the rate of survival of young cancer patients; however, female lymphoma patients frequently face a temporary or permanent loss of fertility when treated with traditional cytotoxic agents. The potential loss of fertility is an important concern that can influence treatment decisions for many premenopausal cancer patients. The negative effect of chemotherapeutic agents and treatment protocols to patients’ fertility–referred to as fertotoxicity–are thus an increasingly important cancer survivorship issue. We have developed a novel nanoscale formulation of arsenic trioxide, a potent drug for treatment of hematological malignancies, and demonstrate that it has significantly better activity in a murine lymphoma model than the free drug. In parallel, we have developed a novel *in vitro* assay of ovarian follicle function that predicts *in vivo* ovarian toxicity of therapeutic agents. Our results reveal that the nanotherapeutic agent is not only more active against lymphoma, but is fertoprotective, i.e., it is much less deleterious to ovarian function than the parent drug. Thus, our *in vitro* assay allows rapid evaluation of both established and experimental anticancer drugs on ovarian reserve and can inform the selection of efficacious and fertility-sparing treatment regimens for reproductive-age women diagnosed with cancer.

## Introduction

According to the National Cancer Institute, 72,000 adolescents and young adults (ages 15–39 years) are diagnosed with cancer each year [1]. The most prevalent types of cancer in this patient population include lymphoma, leukemia, germ cell tumors (including testicular cancer), melanoma, breast, and cervical cancers [1]. Due to advances in anticancer therapy, many of these young people will survive their cancer. Yet many of these life-saving, potent therapies also threaten the future fertility of young cancer patients [2]. Post-treatment fertility is a major concern of young breast cancer patients; in one survey, 29% of these women made cancer treatment decisions based on the fertotoxicity of therapy, yet only 51% felt their concerns were adequately addressed [3].

Many chemotherapeutic agents can damage ovarian tissue and impair follicle function, causing temporary or permanent infertility in female children, adolescents, and young adults [2], [4], [5]. In most clinical studies, amenorrhea is used as a measure of the fertotoxicty of chemotherapeutic agents; however, amenorrhea may not be the best marker of ovarian damage or the risk of future infertility. The most damaging agents to future fertility are those that reduce the ovarian reserve, or the number of ovarian follicles (each of which encloses a single oocyte) that are capable of supporting oocyte growth, maturation, and fertilization. Alkylating agents are known to damage growing oocytes as well as early-stage follicles, causing temporary and sometimes permanent amenorrhea and reduced uterine receptivity [6]–[9]. By comparison, we have limited knowledge about the fertotoxic effects of emerging chemotherapeutics, a gap that presents a major obstacle to the informed selection of fertility-sparing treatment regimens for reproductive-age women or the discussion of options for fertility preservation prior to therapy.

Arsenic trioxide (As_2_O_3_) is an FDA-approved therapeutic agent that has been highly successful in treating acute promyelocytic leukemia [10]–[14] and has shown promise in adult T-cell leukemia/lymphoma [15]. The mechanism of action of As_2_O_3_ is complex, and includes induction of apoptosis by reactive oxygen species, promotion of cellular differentiation, and inhibition of angiogenesis [16], [17]. While As_2_O_3_ has shown promising efficacy in preclinical models of solid tumors, this success has not been replicated in clinical trials due to its rapid renal clearance and dose-limiting toxicities [18], [19]. Recent reports have shown promising efficacy of As_2_O_3_ in both clinical lymphoma specimens and lymphoma cell lines [20]. However activity of As_2_O_3_ in clinical trials has not shown benefit in most subtypes of lymphoma [20]. In order to improve the antitumor activity of As_2_O_3_, a nanoparticulate formulation of As_2_O_3_ was recently developed [21], [22]. In this delivery system, transition metals (e.g., Ni^2+^, Co^2+^, Pt^2+^) are used to stably encapsulate As_2_O_3_ as a nanoprecipitate inside a liposomal vesicle that we termed “nanobins” [NB(Ni,As)]; so named because each vesicle contains many precipitated arsenic-nickel particles. This formulation of As_2_O_3_ has been shown to decrease the plasma clearance of arsenic, improve tumor delivery of arsenic, inhibit triple-negative breast cancer growth and attenuate toxicity *in vitro* [23]. We hypothesized that the nanoparticulate formulation of As_2_O_3_ in NB(Ni,As) would have antitumor activity at lower doses and be less toxic to female reproductive function than free As_2_O_3_.

Despite the clinical use of As_2_O_3_, studies on its effects on reproductive function are focused on ingestion of environmental arsenic and developmental toxicology in rodents [24]–[27]. *In vivo* methods for evaluating the reproductive effects of drugs involve time-consuming testing in animals and are not required for FDA approval of cytotoxic cancer chemotherapeutics [28]. We sought to develop a rapid *in vitro* assay to measure the impact of chemotherapeutic agents on ovarian reserve that could guide the selection of therapies based on the potential for reproductive toxicity. Three assay design criteria were important to meet our goal: 1) the assay must quantify follicle health with low operator expertise; 2) the assay must have a short development time; and 3) the assay method must be scalable to enable high-throughput screening.

We hypothesized that the alginate hydrogel system, originally developed for the three-dimensional culture of isolated ovarian follicles and fertility preservation, provided an assay platform that met these criteria [29]–[31]. Culture of single ovarian follicles within alginate hydrogel essentially recapitulates *in vitro* the follicle growth that occurs *in vivo* within the ovary. Using the alginate hydrogel system, primordial follicles from mice, nonhuman primates [32] and humans [31] have been successfully cultured to produce fully mature oocytes. We have also demonstrated that the mature oocytes from *in vitro* cultured mouse follicles are of good quality, can be fertilized, and result in live births [30]. Importantly, the system is easy to implement and provides rapid assessment of follicle development after treatment with chemotherapeutic agents.

In this study, we first demonstrated that the antitumor efficacy of NB(Ni,As) was superior to free As_2_O_3_ in a murine model of lymphoma, and that NB(Ni,As) was less fertotoxic than free As_2_O_3_ *in vivo*. As reported in previous studies, the increased efficacy and reduced fertotoxicty are likely related to differences in plasma pharmacokinetics, tumor uptake, and systemic biodistribution of the encapsulated and free As_2_O_3_ agents [23]. We then correlated these *in vivo* observations with the results of our novel *in vitro* follicle growth assay. We have now developed an *in vitro* assay that can be scaled up and utilized to evaluate the potential for reproductive side effects, both in early-stage drug development and of existing agents and their combinations. The issue of fertotoxicity of chemotherapeutic agents is important, not only to the research community, but also to pharmaceutical development teams, the oncologists who treat reproductive-age patients, and patients who wish to preserve their fertility and ensure a high quality of life as cancer survivors. Knowledge of the fertotoxicity of chemotherapy regimens is also critical given that several interventions of fertility preservation, such as oocyte retrieval and ovarian tissue banking are more effective in chemotherapy naïve patients.

## Materials and Methods

### Preparation of As_2_O_3_-loaded Nanobins

Arsenic trioxide-loaded nanobins [NB(Ni,As)] and NB(NaCl) were prepared as described previously [23]. Briefly, a dry-lipid film consisting of DSPC (1,2-distearoyl–glycero-3-phosphocholine (Avanti Polar Lipids; Alabaster, AL), DSPE-PEG_2000_ (1,2-Distearoyl-sn-glycero-3-Phosphoethanolamine-N[Methoxy(Polyetheylene glycol)-2000] (ammonium salt) (Avanti Polar Lipids) and Cholesterol (51/4/45 mol %) was hydrated with 300 mM Ni(OAc)_2_ solution for 1 hour at 60°C for 1 h. Arsenic trioxide and cholesterol was obtained from Sigma (St. Louis, MO) and Ni(OAc)_2_ was obtained from Strem Chemical (Newburyport, MA). The lipids were then sequentially extruded through 200 nm and 100 nm polycarbonate membranes (Whatman International, Maidstone, United Kingdom) with a Lipex Extruder (Northern Lipids, Burnaby, BC, Canada) operated at 60°C. Unencapsulated Ni(OAc)_2_ was removed by tangential flow filtration (TFF). The purified vesicles were heated with As_2_O_3_ solution for 2 h at 60°C to generate NB(Ni,As). A second TFF step was then used to remove encapsulated As_2_O_3_. The NB(NaCl) are prepared identically except they are hydrated with 20 mM HEPES, 150 mM NaCl, pH 7.4 and undergo a single TFF purification after extrusion. The concentration of nickel, arsenic and phosphorus was determined by inductively coupled plasma-optical emission spectroscopy (ICP-OES) (Vista MPX, Varian, USA). The size of the particles was determined by dynamic light scattering on a Zetasizer Nano ZS (Malvern Instruments, Malvern, UK).

### Cell Culture and Annexin-V Assays

L540 [33] and RAMOS [34] cells were obtained from the Tumor Biology Core Facility of the Northwestern University Robert H. Lurie Cancer Center and Z138 cells [35] were a gift from Dr. Steven Rosen. L540 and RAMOS cells were maintained in Roswell Park Memorial Institute medium (RPMI 1640, Invitrogen Corporation, Carlsbad, CA) and Z138 cells were maintained in Iscove’s Modified Dulbecco’s Medium (IMDM, Invitrogen), and all media was supplemented with 2 mM L-glutamine and 10% fetal bovine serum (FBS, Invitrogen). The cells were grown in an incubator at 37°C with 5% CO_2_. 1×10^6^ cells were plated in 12 well plates and treated with 0.5, 5 or 50 µM [As] of As_2_O_3_, NB(Ni,As) or NB(NaCl), equivalent phospholipid to the NB(Ni,As), for 18 hours. The media was then removed and the cells were washed in ice cold phosphate buffered saline. The cells were resuspended in annexin biding buffer (10 mM HEPES, 140 mM NaCl, 2.5 mM CaCl_2_, Invitrogen) containing 10 µg/ml DAPI (4′,6-diamidino-2-phenylindol) and Alexa Fluor® 647 Annexin V (Invitrogen) according to manufactures instructions. Control cells were prepared without the annexin label. The cells were then analyzed on a BD LSR II Flow Cytometer. The data was analyzed with FlowJo (Tree Star, Ashland, OR).

### Ethics Statement

Female Rag2M mice (British Columbia Cancer Research Center Animal Resource Center, Vancouver, British Colombia, Canada) were utilized for the xenograft experiment at the BC Cancer Agency (Vancouver, BC). The xenograft experimental design and protocol were reviewed and approved by the Institutional Animal Care Committee (IACC) at the University of British Colombia prior to conducting the studies (protocol #A05-158), and were performed in accordance with the Canadian Council on Animal Care Guidelines.

Female CD1 mice (7.5-week-old; Harlan, Indianapolis, IN) were used for *in vivo* toxicity studies, and ovaries and immature follicles isolated from prepubertal, 12- to 14-day-old female CD1 mice were used in *in vitro* toxicity assays. Mice were treated in accordance with the NIH Guide for the Care and Use of Laboratory Animals, and protocols were approved by the IACUC at Northwestern University (protocol# 2012-1181).

### Z138C Xenograft Experiment

The Z138C human B-cell lymphoma mantle cell line was obtained from the Martin Dyer lab (University of Leicester, UK) and tested negative for mycoplasma. Cells were grown in RPMI 1640 supplemented with 2 mM L-glutamine and 10% FBS at 37°C in 5% CO_2_. Cells from passages 3 to 10 were grown to 80%–90% confluence and harvested for implantation. Cells were resuspended in cold growth medium at 50×10^6^ cells/ml and then mixed on ice with Matrigel (1∶1). A 2×2-cm patch of hair was removed from the lower back region of 72 female Rag2M mice with hair clippers, and 100 µl (5×10^6^ cells) of the Z138C cell-Matrigel mixture was implanted subcutaneously using a 27-gauge needle. After 16 days, the mice were staged. Starting on post-implantation day 18, mice were treated weekly for up to 1 month with PBS or 4, 6, or 8 mg/kg As_2_O_3,_ equivalent molar dose of arsenic as NB(Ni,As) and equivalent phospholipid concentration of NB(NaCl), by intravenous injection in the tail vein using a 28-gauge needle (n = 8 for each treatment group). Injection volume was 200 µL/20 g mouse body weight. Tumor growth was monitored by measuring tumor dimensions with calipers starting the first day of treatment and then 3 times per week thereafter. Tumor volumes were calculated according to the equation (L×W×H)/2, with the length (mm) being the longest axis of the tumor. Animals were also weighed at the time of tumor measurement. Tumors were allowed to grow to a maximum of 1000 mm^3^, at which point the animal was sacrificed.

### Cyclicity Studies and Isolation of Mouse Ovaries and Follicles

Animals were housed in a temperature- and light-controlled environment (12 L:12 D) and provided with food and water *ad libitum*. CD1 mice were fed Harlan Teklad Global irradiated 2919 chow, which does not contain soybean or alfalfa meal and therefore contains minimal phytoestrogens.

CD1 females (N = 40) were purchased at 6 weeks of age, housed in pairs, and allowed to acclimate for 1.5 weeks. The animals were checked for cyclicity by vaginal lavage and cytological analysis starting at 7.5 weeks of age, and cycling data were recorded. Once the animals had 2 complete cycles (approximately 2 weeks), they were randomly assigned to a treatment group [n = 10 for each group: PBS or 4 mg/kg NB(NaCl), As_2_O_3_, or NB(Ni,As)], and received 100-µl intraperitoneal injections twice weekly for 3.5 weeks. Cycling was monitored during treatment and then continued to 5.5 weeks, at which time the animal was sacrificed. The ovaries, uterus, kidneys, liver, sedimented red blood cells, and plasma were collected from each animal. Ovaries and uteri were fixed and embedded in paraffin, sectioned, and stained with hematoxylin and eosin to examine histology. Blood and plasma samples were collected using an IACUC-approved exsanguination procedure.

### Plasma Arsenic Pharmacokinetic Analysis

Six-week-old female CD1 mice (N = 36) were housed in groups of 3 and allowed to acclimate for 1.5 weeks. After confirming normal cyclicity for 2 weeks by vaginal lavage, the mice were randomized into 2 treatment groups (n = 18 in each group) and injected with 4 mg/kg of As_2_O_3_ or NB(Ni,As). All animals were in either metestrus or diestrus at the time of injection. After injection, As_2_O_3_-treated mice were sacrificed at 2, 4, 6, 12, 24, and 48 hours (n = 3 for each time point) and NB(Ni,As)-treated mice were sacrificed at 2, 6, 12, 24, 36, and 48 hours (n = 3 for each time point). The liver, kidneys, ovaries, uterus, and plasma were immediately collected and stored at −80°C. Arsenic uptake in each tissue was measured by inductively coupled plasma-mass spectrometry (ICP-MS), and pharmacokinetic analysis was performed using the SAAM II software system (SAAM Institute, Seattle, Washington).

### Inductively Coupled Plasma-mass Spectrometry

Tissue arsenic levels were determined by ICP-MS with a Thermo X Series II inductively coupled plasma-mass spectrometer (Thermo-Fisher, Waltham, MA). Samples were prepared by digesting tumors in 500 µl concentrated trace metal-free grade nitric acid (69%) in capped, metal-free falcon tubes for 2 hours at 60°C. At 20-minute intervals during the digestion, the sample tubes were vortexed and vented in a fume hood. After 2 hours, the digests were filtered through a 0.45-µm polytetrafluoroethylene (PTFE) filter into a fresh metal-free falcon tube. For ICP-MS analysis, a portion of the filtered digest was diluted with ultrapure laboratory grade water (18 Ω) and an internal standard mixture of Sc, Tb, Y, In, and Bi (CPI International, Santa Rosa, CA) was added. Standards between 0 and 90 ppb were made using a custom mixed element solution (CPI International). The final ICP-MS samples and elemental standards were prepared in a matrix of 2% nitric acid containing 0.1 Triton X-100 and 5% acetic acid.

### *In Vitro* Follicle Culture and Toxicity Assay

Ovaries were isolated from 12- to 14-day-old CD1 mice into prewarmed collection media (Liebovitz L-15, Invitrogen) containing 1 mg/ml bovine serum albumin (BSA) and 50 IU/ml penicillin/streptomycin (Invitrogen). Two ovaries were incubated per culture plate. A total of 45 ovaries were used for ICP-MS and 20 ovaries were used for follicle isolation. Early secondary follicles (oocytes surrounded by 2–3 granulosa cell layers) were isolated from the ovaries, and whole ovaries and isolated follicles were transferred to α-MEM containing 1 mg/ml BSA, 5 µg/ml insulin, 5 µg/ml transferrin, and 5 ng/ml sodium selenite, and incubated at 37°C at 5% CO_2_ on a horizontal shaker (Figure S1). After 3 hours, 250 µl of PBS or 3, 30, or 90 µM As_2_O_3_, NB(NaCl), or NB(Ni,As) was added to the cultures; after 3 hours, the ovaries and follicles (25 per treatment group and 4 repeats) were washed in media 3×10 minutes. Isolated follicles were then encapsulated into sterile 0.5% (w/v) alginate beads as described previously [30]. Encapsulated follicles were grown for 10 days in α-MEM containing 1 mg/ml bovine fetuin (Sigma-Aldrich, St. Louis, MO), 5 µg/ml insulin, 5 µg/ml transferrin, 5 ng/ml sodium selenite (Sigma-Aldrich), 3 mg/ml BSA, and 10 mIU rhFSH (gift from Organon, Roseland, NJ).

### Statistics

Tumor volume, arsenic concentration, and follicle growth and survival data were subjected to one-way analysis of variance followed by Dunnett’s or Bonferroni’s multiple comparison post-hoc test to determine the significance of differences between each treatment group using Prism 4 (GraphPad Software, Inc.). Calculated values are shown as mean ± SEM with a significance level of *P*<0.01 being considered statistically significant, unless otherwise noted.

## Results

### As_2_O_3_ is Cytotoxic to Lymphoma Cell Lines *in vitro* and NB(Ni,As) Inhibits Z138C Lymphoma Xenograft Tumor Growth *in vivo*

Hodgkin’s lymphoma and Burkitt’s lymphoma are highly prevalent in patients of reproductive age [36]. We evaluated the *in vitro* induction of apoptosis of As_2_O_3_ and NB(Ni,As) in L540 Hodgkin’s lymphoma, RAMOS Burkitt lymphoma, and Z138C mantle cell lymphoma cell lines. We determined that free As_2_O_3_ induces apoptotic cell death in all 3 cell lines, while free NB(Ni,As) and NB(NaCl) (a vehicle control nanobin) have minimal cytotoxicity in this 18 hour assay (Figure S2). We have observed a similar effect in breast, ovarian, SUDHL-4 lymphoma cells and all of the other cancer cell lines that we have evaluated to date [21]–[23]. The attenuation of cytotoxicity is based on the fact that encapsulated arsenic is not bioactive until it is released, which occurs over a 48 h period [23].

**Figure 1:**
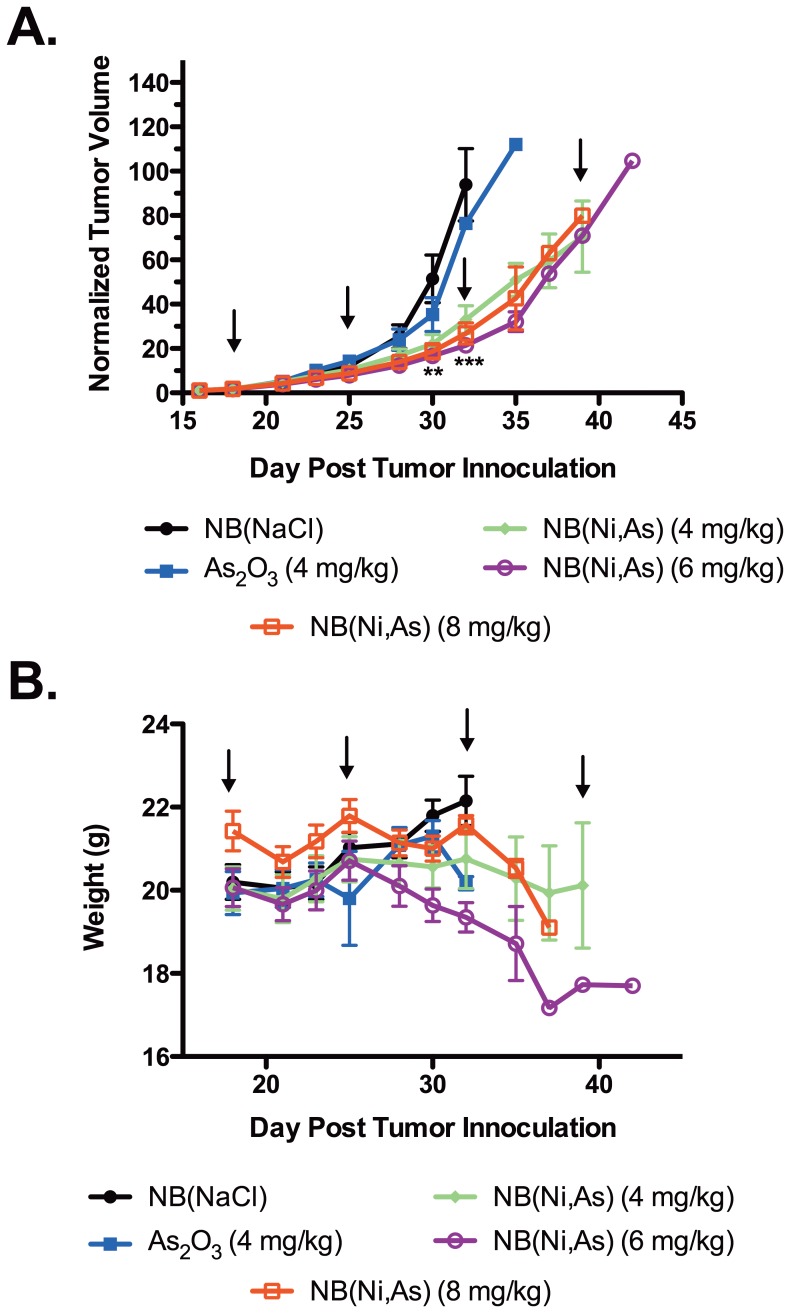
NB(Ni,As) inhibits mantle cell lymphoma growth. 18 days after inoculation with Z138C lymphoma cells, Rag2M mice were randomized and treated with weekly injections of NB(NaCl), As_2_O_3_ (4, 6, or 8 mg/kg), or NB(Ni,As) (4, 6, or 8 mg/kg). (A) Tumors treated with NB(Ni,As) were significantly smaller than those treated with NB(NaCl). **, *P*>0.01, ***, *P*>0.001. Arrows indicate treatment. (B) Weight was monitored daily during the treatment period. Injection of As_2_O_3_ was acutely toxic, whereas mice injected with NB(NaCl) showed normal weight gain. Mice injected with any dose of NB(Ni,As) lost weight, though mice treated with 4 mg/kg showed the least amount of weight loss during the treatment period.

In order to evaluate the *in vivo* activity of NB(Ni,As) we chose to utilize the Z138C mantle cell lymphoma subcutaneous xenograft model in Rag2M mice. We chose to use the Z138C cells since the Z138C and L540 cell lines had similar cytotoxicity *in vitro* and our previous experience with the Z138C model [35]. Mice bearing Z138C lymphoma xenograft tumors were treated weekly with either NB(NaCl), As_2_O_3_ (4, 6, or 8 mg/kg), or NB(Ni,As) (4, 6, or 8 mg As_2_O_3_/kg) for 1 month. Free As_2_O_3_ at 4 mg/kg was not effective in slowing tumor growth on this schedule and was equivalent to the control NB(NaCl), whereas the 6 and 8 mg/kg doses of As_2_O_3_ were acutely toxic to the mice (data not shown). By contrast, NB(Ni,As) effectively inhibited tumor growth at all 3 doses tested compared with NB(NaCl) and free As_2_O_3_ (Figure 1A). The 4 mg/kg dose of NB(Ni,As) and As_2_O_3_ were well tolerated by the mice, with no signs of toxicity (<15% weight loss). Treatment with 6 mg/kg and 8 mg/kg of NB(Ni,As) resulted in weight loss (Figure 1B). Thus, NB(Ni,As) at 4 mg/kg showed antitumor efficacy with minimal effect on weight in an animal model of lymphoma.

### NB(Ni,As) Limits Systemic Exposure of Arsenic Compared with Free As_2_O_3_

**Figure 2:**
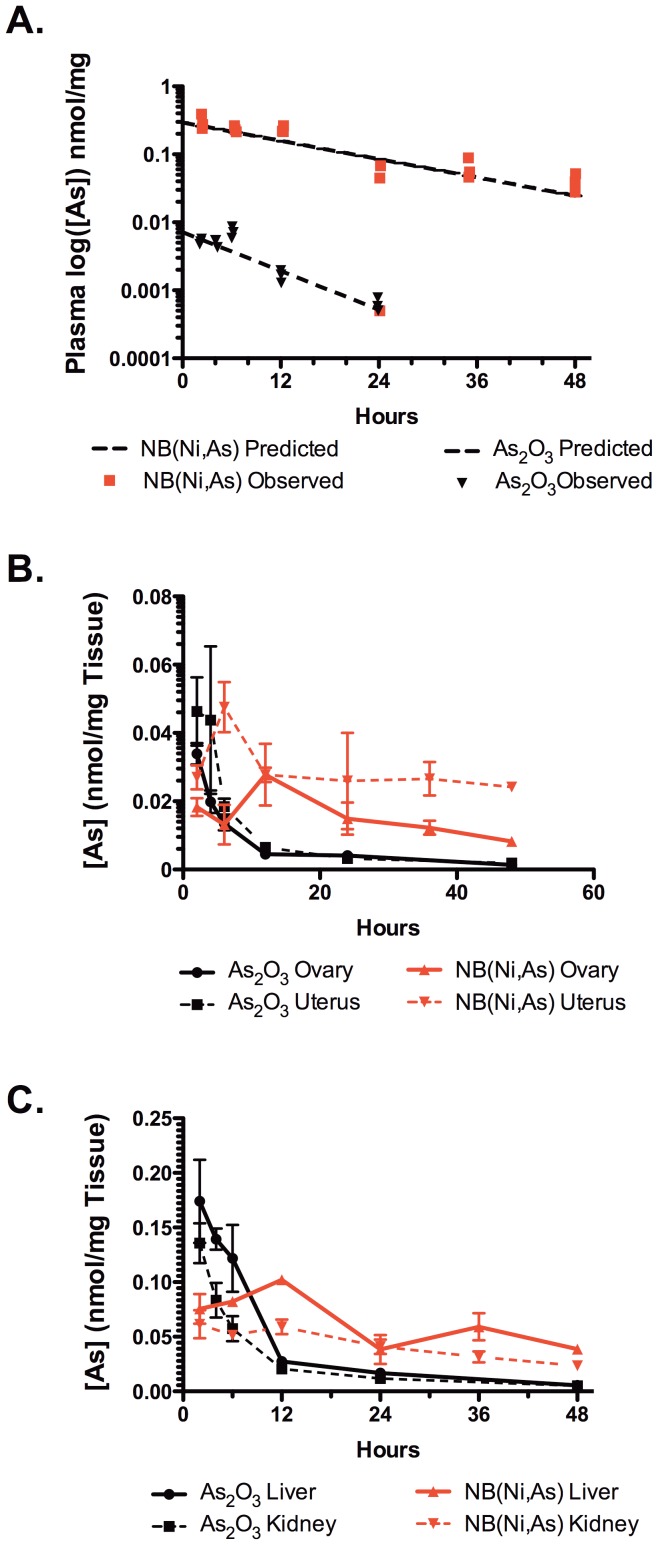
Arsenic plasma concentrations and uptake in mouse tissues and cultured mouse ovaries. (A) NB(Ni,As)-treated (4 mg/kg) mice had reduced clearance of arsenic in plasma and increased peak plasma concentration compared with As_2_O_3_-treated (4 mg/kg) mice. (B) Arsenic levels in the uterus and ovaries peaked and cleared more rapidly in mice treated with As_2_O_3_ compared with mice treated with NB(Ni,As). (C) Arsenic levels in the liver and kidney paralleled those in the uterus. Error bars represent ± SEM.

We next examined the plasma pharmacokinetics and biodistribution of NB(Ni,As) and As_2_O_3_ *in vivo*. A single 4 mg/kg dose of As_2_O_3_ or NB(Ni,As) was administered to female CD1 mice by intraperitoneal injection. Mice were sacrificed at various time points up to 48 hours after injection, and the liver, kidneys, ovaries, uterus, and plasma were collected. Total arsenic concentration was measured by ICP-MS (Figure 2). Pharmacokinetic analysis of plasma arsenic levels revealed that plasma elimination clearance and the steady-state volume of distribution (V_ss_) of As_2_O_3_ were much higher than those of NB(Ni,As). The elimination clearance of NB(Ni,As) was 0.18 ml/hr while that of As_2_O_3_ was 15.2 ml/hr. Furthermore, the V_ss_ of NB(Ni,As) (3.5 ml) was similar to the predicted plasma volume of a mouse, suggesting that NB(Ni,As) largely confines arsenic to the intravascular space. By contrast, the V_ss_ of As_2_O_3_ (139.2 ml) indicated rapid, extensive, nonselective tissue distribution. These pharmacokinetic parameters are consistent with single-dose plasma pharmacokinetic analysis of free As_2_O_3_ and NB(Ni,As) in rats [23].

The peak concentration of arsenic in the ovary was higher in the As_2_O_3_ group than in the NB(Ni,As) group (Figure 2B), which is consistent with rapid and extensive distribution of free As_2_O_3_. However, the actual peak concentration of arsenic in the ovary was likely missed in As_2_O_3_-treated animals because distributional equilibrium had been reached by the time the first tissue samples were collected. The arsenic level in mice treated with NB(Ni,As) persisted longer in the ovary; however, this measurement likely included arsenic in the vascular space of the ovary. The concentration of arsenic in the uterus paralleled that of the ovary, although total arsenic exposure in the uterus was higher than in the ovary, which is consistent with greater uterine vascularity compared with the ovary (Figure 2B).

Peak arsenic concentrations were higher in the liver and kidneys of As_2_O_3_-treated mice compared with NB(Ni,As)-treated mice, but the arsenic concentration dropped rapidly over the course of 48 hours in these organs, consistent with the rapid plasma clearance of free As_2_O_3_ (Figure 2C). Levels of arsenic in the liver and kidneys of mice treated with NB(Ni,As) fell slowly during the 48 hour time period due to the extended plasma half-life and reduced clearance of NB(Ni,As) compared with As_2_O_3_ (Figure 2C).

### Impact of As_2_O_3_ and NB(Ni,As) on Ovarian Reserve in Mice

**Figure 3:**
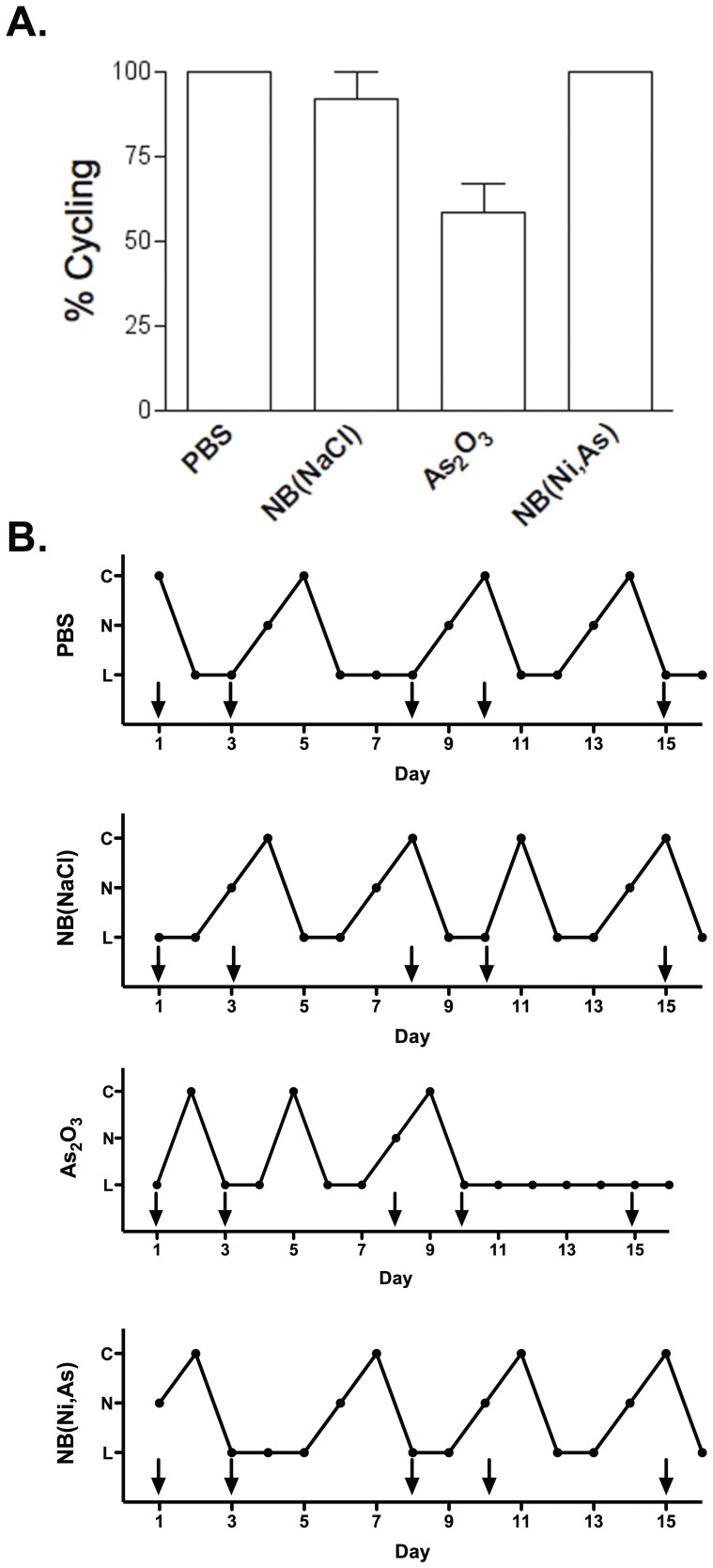
Effect of As_2_O_3_ and NB(Ni,As) on ovarian cyclicity. (A) All mice treated with PBS or NB(Ni,As) displayed normal cyclicity. Estrus cycles were stopped in 40% of mice treated with 4 mg/kg As_2_O_3_. One mouse treated with NB(NaCl) skipped one cycle, but otherwise cycled normally. Error bars represent ± SEM. (B) Representative cycles for each treatment group, [**C**, estrus, cornified epithelium present], [**L**, metestrus/diestrus, leukocytes present], [**N**, proestrus, nucleated cells present]. Arrows indicate treatment.

To assess the effect of repeat treatments of As_2_O_3_ and NB(Ni,As) on female reproductive function *in vivo*, mice were treated with PBS or 4 mg/kg As_2_O_3_, NB(NaCl), or NB(Ni,As) twice a week for 3.5 weeks. The dose and schedule were chosen based on demonstrated both efficacy and minimal toxicity in both the breast cancer and lymphoma models [23]. Prior to treatment, all mice were cycling regularly (3- to 4-day cycles) for 2 full weeks as determined by vaginal lavage and endometrial cytology examination. During the course of treatment, the estrus cycle was determined daily as a measure of ovarian function. All of the mice injected with PBS showed normal estrus cyclicity (Figure 3A). By contrast, 40% of mice injected with As_2_O_3_ stopped cycling within 10 days of the initiation of treatment, and mice in the As_2_O_3_ group had shortened cycles (2–3 days; Figure 3A, B). Mice injected with NB(NaCl) showed 90% normal cyclicity (Figure 3A); 1 mouse missed a single cycle but resumed cycling and continued to do so normally until the end of the study. Notably, mice treated with NB(Ni,As) displayed normal cyclicity throughout treatment (Figure 3A, B). Thus, NB(Ni,As) had a lower fertotoxic effect than free As_2_O_3_ *in vivo*.

**Figure 4:**
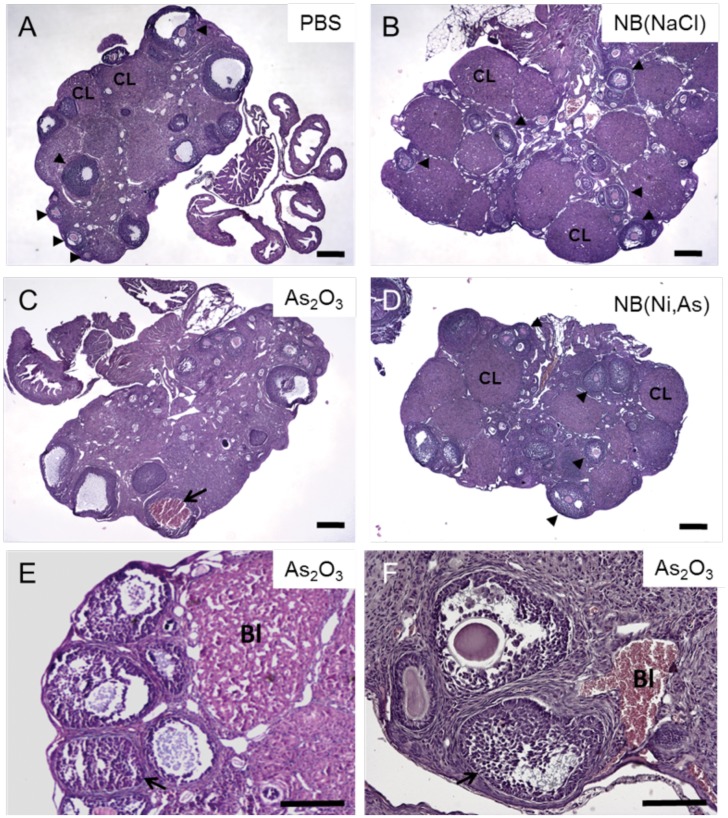
Effect of As_2_O_3_ and NB(Ni,As) on ovarian histology. Hematoxylin and eosin staining of ovarian sections from mice following 3.5-weeks of treatment with (A) PBS (4× magnification); (B) 4 mg/kg NB(NaCl) 4×magnification); (C, E, F) 4 mg/kg As_2_O_3_ (4× and 10× magnification); or (D) 4 mg/kg NB(Ni,As) (4× magnification). (A, B, D) Ovaries from PBS-, NB(NaCl)-, and NB(Ni,As)-treated mice show normal ovarian histology and contain follicles of all stages as well as corpora lutea. (C, E, F) Ovaries isolated from As_2_O_3_-treated mice contained blood filled cysts and leaky vasculature. Measurement bars represent 100 µm (A–D) and 200 µm (E, F). Follicles are indicated with arrowheads and corpora lutea are labeled “CL.” Blood-filled cysts are indicated with arrows and areas of leaky vasculature are labeled “Bl” in panels C, E, and F.

At the termination of the *in vivo* cyclicity study, the arsenic concentration in 2 sets of ovaries from each treatment group was measured. The ovaries of mice exposed to As_2_O_3_ contained 0.05 nmol/mg arsenic, whereas ovaries exposed to NB(Ni,As) contained 0.02 nmol/mg arsenic (Figure S3). The remaining ovaries were fixed and processed for histologic analysis. Mice treated for 3.5 weeks with PBS or NB(NaCl) had normal ovaries that contained follicles at all stages of development, from the primordial through the antral follicle stage, as well as the presence of corpora lutea (Figure 4A, B). Ovaries from mice treated with 4 mg/kg As_2_O_3_ showed blood filled cysts (Figure 4C, E) and areas of hemorrhage (Figure 4F). By contrast, mice treated with 4 mg/kg NB(Ni,As) showed normal ovarian histology (Figure 4D). These histologic data confirmed the observed physiologic effects of NB(Ni,As) and As_2_O_3_ on reproductive cyclicity in mice.

### *In vitro* Assay of As_2_O_3_ and NB(Ni,As) on Follicle Development

**Figure 5:**
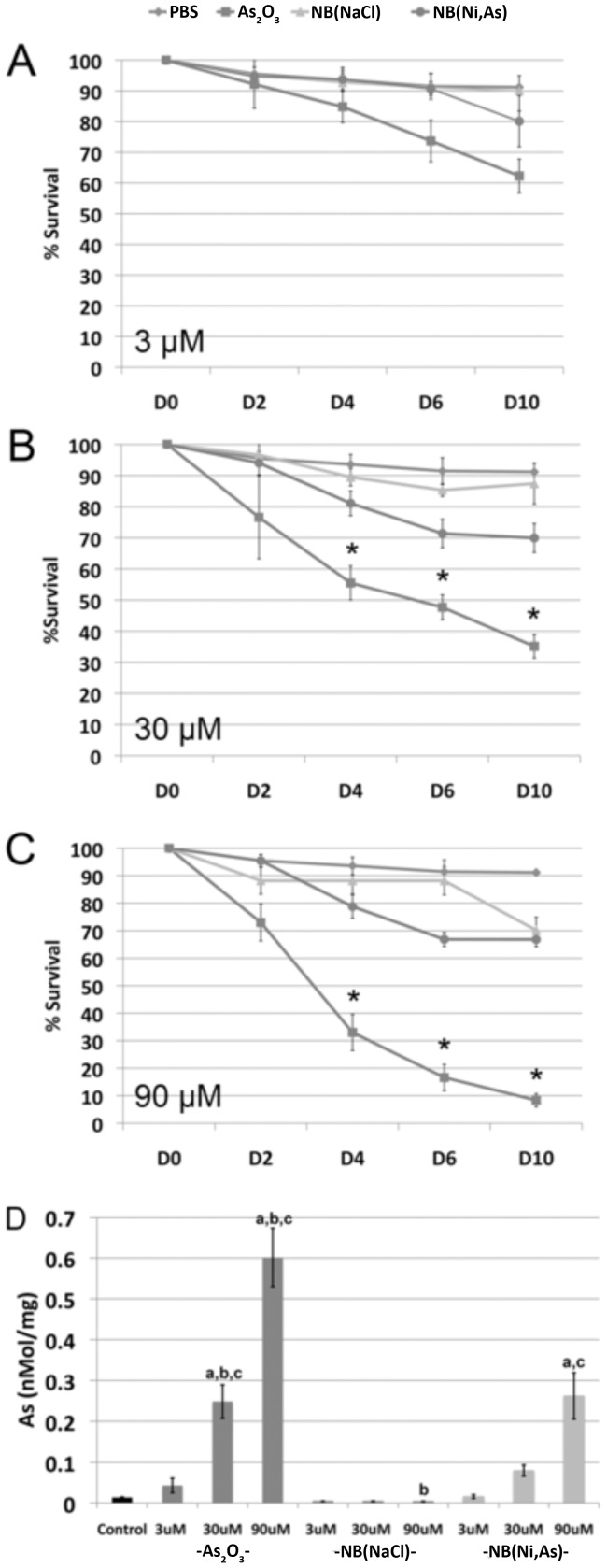
Follicle survival after *in vitro* arsenic exposure. Isolated ovarian follicles were incubated in PBS or (A) 3, (B) 30, or (C) 90 µM As_2_O_3_, NB(NaCl), or NB(Ni,As) for 3 hours. Individual follicles were then encapsulated in alginate and cultured for 10 days to analyze survival rate. (A) At 3 µM As_2_O_3_, follicle survival was not statistically significantly different compared with PBS, NB(NaCl), or NB(Ni,As). (B) At 30 µM As_2_O_3_, follicle survival was significantly less starting at day 4. C, At 90 µM As_2_O_3_, follicle survival dropped to 30% by day 4. At all concentrations, NB(Ni,As)-treated follicle survival was not significantly different than that of PBS or NB(Ni,As). (D) Ovaries were incubated in PBS or 3, 30, or 90 µM As_2_O_3_, NB(NaCl), or NB(Ni,As) for 3 hours. Arsenic content in the cultured ovaries was examined by ICP-MS. Arsenic content was significantly higher in As_2_O_3_-treated ovaries than in PBS-, NB(NaCl)-, or NB(Ni,As)-treated ovaries at 30 and 90 µM. Arsenic content in NB(NaCl)-treated ovaries was only significantly less than in ovaries treated with the highest dose of NB(Ni,As) (90 µM). (a) is *P*<0.01 compared with PBS, (b) is *P*<0.01 compared with NB(Ni,As) at the same concentration, (c) is *P*<0.01 compared with NB(NaCl) at the same concentration. Error bars represent ±SEM.

To validate our novel *in vitro* follicle-based assay, individual follicles were isolated from the ovaries of 12- to 14-day-old mice and treated in culture for 3 hours with PBS, NB(NaCl), As_2_O_3_ (3, 30, or 90 µM As) or NB(Ni,As) (3, 30, or 90 µM As). Each follicle was then encapsulated in alginate hydrogel matrix and cultured for 10 days to assess the effect of arsenic exposure on follicle survival and growth. Survival of follicles treated with PBS or NB(NaCl) vehicle was similar for all 3 treatment concentrations (Figure 5A-C). As_2_O_3_ treatment at doses as low as 3 µM had a detrimental effect on follicle survival by day 6 (Figure 5A), and the majority of follicles treated with 90 µM As_2_O_3_ died by day 4 (Figure 5C). Follicles treated with 3 µM NB(Ni,As) initially survived at rates similar to those of follicles treated with either PBS or NB(Ni,As) (Figure 5A), but survival of follicles treated with either 30 µM or 90 µM NB(Ni,As) was only 70% on day 6 (Figure 5B, C). Arsenic content in whole ovaries cultured for 3 hours in PBS or 3, 30, or 90 µM As_2_O_3_, NB(NaCl), or NB(Ni,As) was also determined by ICP-MS (Figure 5D). Ovaries treated with NB(Ni,As) contained significantly lower amounts of arsenic compared with ovaries treated with As_2_O_3_.

**Figure 6:**
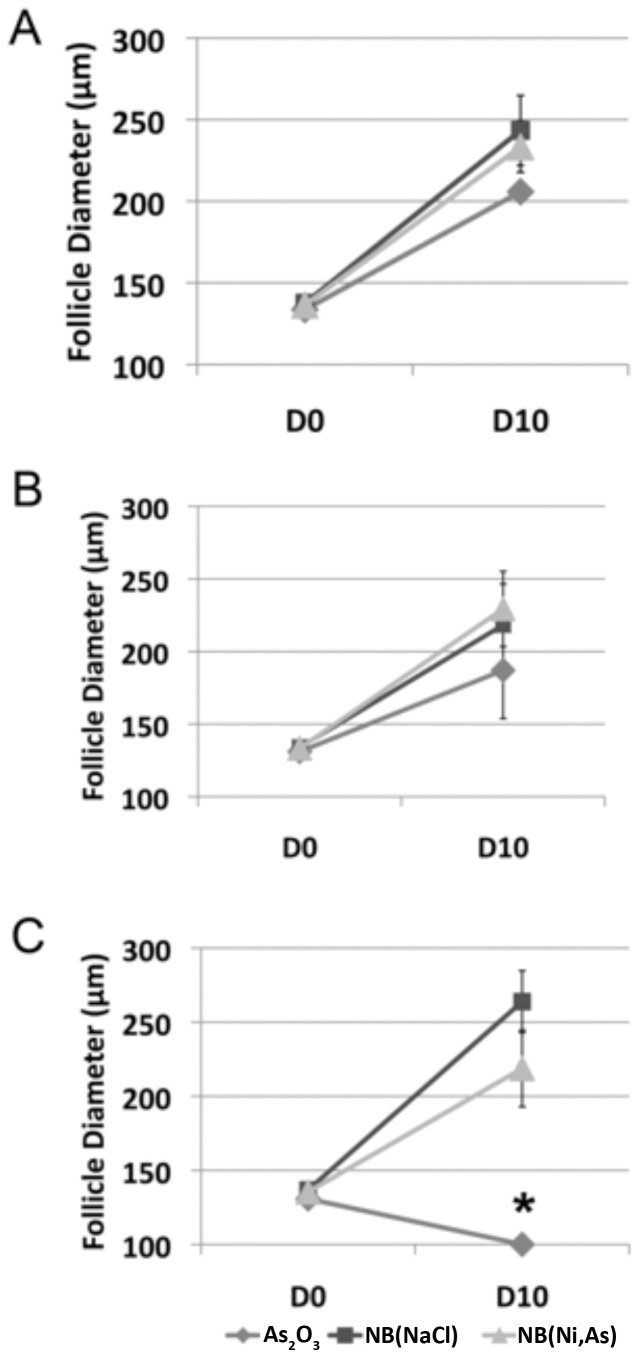
Follicle growth after *in vitro* arsenic exposure. Isolated ovarian follicles were incubated in PBS or (A) 3, (B) 30, or (C) 90 µM As_2_O_3_, NB(NaCl), or NB(Ni,As) for 3 hours. Individual follicles were then encapsulated in alginate and cultured for 10 days to analyze follicle growth. (A, B) At 3 and 30 µM, all surviving follicles grew to approximately the same size, between 200 and 250 µm. (C) At 90 µM, As_2_O_3_-treated follicles showed a decrease in follicle diameter over 10 days of culture, producing significantly smaller follicles than those treated with NB(NaCl) or NB(Ni,As). Error bars represent ± SEM. Asterisk represents significance of *P*<0.05.

Follicles treated with any dose of NB(NaCl) grew to a mean diameter of approximately 250 µm (Figure 6A–C). By contrast, follicles treated with 3 or 30 µM As_2_O_3_ did not grow larger than 200 µm (Figure 6A, B), and those exposed to 90 µM As_2_O_3_ actually decreased in diameter from baseline to 100 µm, indicating follicle death (Figure 6C). Follicles exposed to any concentration of NB(Ni,As), however, showed growth similar to that seen with NB(NaCl), to approximately 250 µm (Figure 6A–C).

## Discussion

Using traditional *in vivo* assays and a novel *in vitro* assay of reproductive (ovarian) function to assess the reproductive toxicity of chemotherapeutic agents, we have determined that nanoscale encapsulation of a potent cytotoxic drug, As_2_O_3_, can provide significant protection of fertility while maintaining or improving the efficacy of the free drug. Using these assays, we demonstrated that arsenic administered as NB(Ni,As) has a lower effective dose and is less toxic than free arsenic to ovarian and follicle function. The efficacy of NB(Ni,As) has been demonstrated in murine models of both triple negative breast cancer and a model of lymphoma, two common cancers in females of reproductive age [23]. In addition, our data showed a dose response to As_2_O_3_, consistent with that reported by Griffin et al in syngetic murine tumor medels [37]. In these studies, As_2_O_3_ as low as 2 mg/kg, induced significant disruption of tumor vasculature, with or without hyperthermia. These studies and our data suggests that delivery of cancer drugs via nanoscale carriers has the potential to increase efficacy and mitigate the impact of cytotoxins on the female reproductive tract by redistributing the agent to the intravascular space. Until the nanoparticle reaches the tumor or is cleared by the reticuloendothelial system, the drug is mostly sequestered inside the vesicle and is not bioactive. This reduces drug exposure to healthy tissues and helps limit systemic toxicities.

Currently, there are several FDA approved nanoformulations approved for cancer therapy [nab-paclitaxel, American BioScience, Inc. [38]; and liposomal pegylated doxorubicin, Ortho Biotech Products L.P. [39]]. Recent reports suggest that liposomal pegylated doxorubicin does not affect the estrus cycle in mice, while it is widely recognized that the parent doxorubicin is highly detrimental to the ovary [40]. Thus, screening of new cancer therapies and new formulations for their potential as fertility sparing therapies is urgently indicated.

Though arsenic accumulated in the ovary *in vitro* and *in vivo* upon treatment with NB(Ni,As), the nanobin encapsulation sequesters the arsenic, thereby limiting its tissue distribution and lessening its impact on ovarian and follicular function. Toxic effects are often correlated to the peak drug levels, and we found higher peak arsenic levels in the ovaries and follicles of animals that were treated with As_2_O_3_ than in those treated with NB(Ni,As). This finding is consistent with the higher toxicity of As_2_O_3_ compared with NB(Ni,As). Systemic treatment with As_2_O_3_ resulted in the formation of bloody ovarian cysts and disrupted the estrus cycle in mice, effects that were not seen with NB(Ni,As) treatment. Arsenic has been shown to regulate steroid receptors such as the androgen receptor, which has been linked to ovarian dysfunction, manifested by the development of polycystic ovaries [36]. Aberrant regulation of steroid receptors in the ovary may have contributed to the observed cyst formation. Future studies will investigate the mechanism of action of arsenic trioxide on ovarian function in order to better understand its effects and develop new approaches to reducing its fertotoxicity when used as an anticancer agent.

Most importantly, our findings concerning the impact of As_2_O_3_ and NB(Ni,As) on ovarian and reproductive function *in vivo* were corroborated in our novel *in vitro* follicle toxicity assay. The follicle is considered to be the functional unit of the ovary; its growth and development are strictly regulated by various growth factors, hormones, and cellular interactions to permit the cyclical production of mature oocytes that are competent to undergo ovulation and fertilization [1]. The availability of three-dimensional follicle culture systems allowed us to evaluate specifically the toxicity of As_2_O_3_ and NB(Ni,As) on ovarian follicle viability and development *in vitro*. Compared with As_2_O_3_, NB(Ni,As)-treated follicles had higher survival and growth rates in our follicle-based assay system.

This is the first report to demonstrate the capacity of an *in vitro* assay to assess the effect of chemotherapeutic agents on ovarian follicle function, and may be useful for estimating the potential impact of chemotherapies or combination regimens on the future fertility of young female cancer patients. More information about the fertotoxicity of agents used to treat cancer is needed as a greater number of young patients with cancer are surviving their disease and may wish to preserve their fertility. Integration of *in vitro* assays of ovarian and follicle health into the preclinical evaluation process for new chemotherapeutic agents is essential in order to determine the potential risks of these agents to fertility and allow patients and their doctors to make more informed decisions about selecting a therapeutic regimen. Finally, this *in vitro* methodology may be adapted to a high-throughput assay to facilitate rapid and cost-effective evaluation of chemotherapeutic agents and delivery systems that limit the risk of infertility secondary to chemotherapy in young women with cancer. Future studies will focus on validation of this assay system in human follicles and determination of fertotoxicity thresholds that correlate with patient fertility outcomes following chemotherapy.

## Supporting Information

Figure S1 ***In vitro*** **follicle-based assay for assessment of chemotherapeutic agent fertotoxicity.** Ovaries are removed and early secondary follicles (oocytes surrounded by 2–3 granulosa cell layers) are isolated and transferred to culture medium. After 3 hours in culture, the follicles are treated with chemotherapeutic agent (or vehicle), then washed and encapsulated into sterile 0.5% (w/v) alginate beads. Encapsulated follicles are cultured *in vitro* for 10 days, then removed from the alginate beads and assessed for follicle and oocyte survival, growth, and morphology; cumulus expansion and antrum formation; steroidogenic capacity; and oocyte meiotic status and capacity for *in vitro* fertilization.
(TIFF)

Figure S2 **Induction of apoptosis by As_2_O_3_, NB(NaCl), or NB(Ni,As).** Apoptosis was measured by staining cells with Annexin V and DAPI after treatment with 0.5, 5.0 or 50 µM As_2_O_3_, NB(NaCl), or NB(Ni,As) for 18 hr. As_2_O_3_ induces apoptosis in Z138(A), L540 (B) and RAMOS (C), while NB(Ni,As) and NB(NaCl) did not. Representative results from multiple trials.
(TIFF)

Figure S3 **Cumulative uptake of arsenic in ovaries treated with As_2_O_3_ and NB(Ni,As).** Ovaries isolated from mice treated with 4 mg/ml As_2_O_3_ or NB(Ni,As) for 3.5 weeks were analyzed for arsenic content by ICP-MS. Ovaries from mice treated with NB(Ni,As) showed significantly less arsenic uptake compared with ovaries from mice treated with As_2_O_3_. Asterisks represent *P*<0.01, error bars represent ± SEM.
(TIF)
