# Supplementary figures and images for: Nano-Encapsulation of Arsenic Trioxide Enhances Efficacy against Murine Lymphoma Model while Minimizing Its Impact on Ovarian Reserve In Vitro and In Vivo
Source: PLoS One. 2013 Mar 20;8(3):e58491. doi: 10.1371/journal.pone.0058491 (PMC3603968; doi:10.1371/journal.pone.0058491)

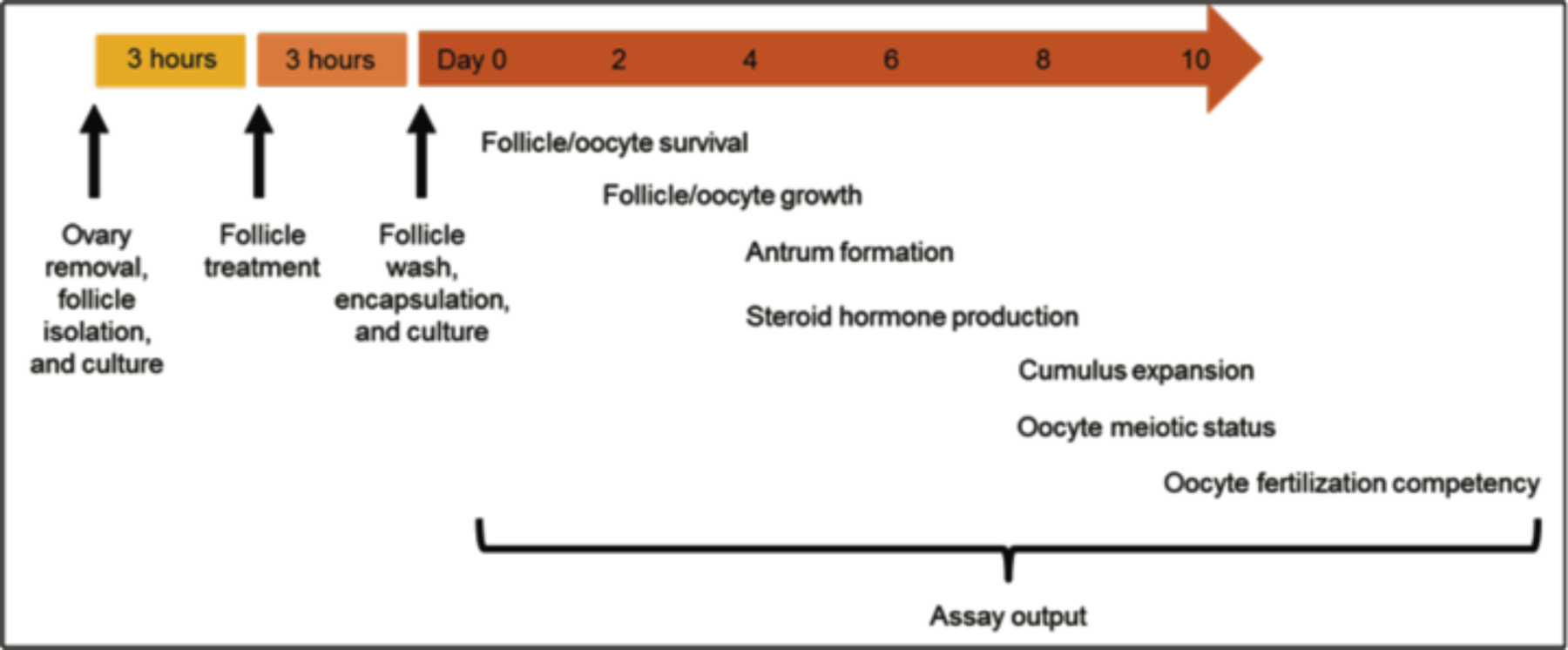

Supplement: Figure S1 — In vitro follicle-based assay for assessment of chemotherapeutic agent fertotoxicity. Ovaries are removed and early secondary follicles (oocytes surrounded by 2–3 granulosa cell layers) are isolated and transferred to culture medium. After 3 hours in culture, the follicles are treated with chemotherapeutic agent (or vehicle), then washed and encapsulated into sterile 0.5% (w/v) alginate beads. Encapsulated follicles are cultured in vitro for 10 days, then removed from the alginate beads and assessed for follicle and oocyte survival, growth, and morphology; cumulus expansion and antrum formation; steroidogenic capacity; and oocyte meiotic status and capacity for in vitro fertilization. (TIFF) [file pone.0058491.s001.tif]

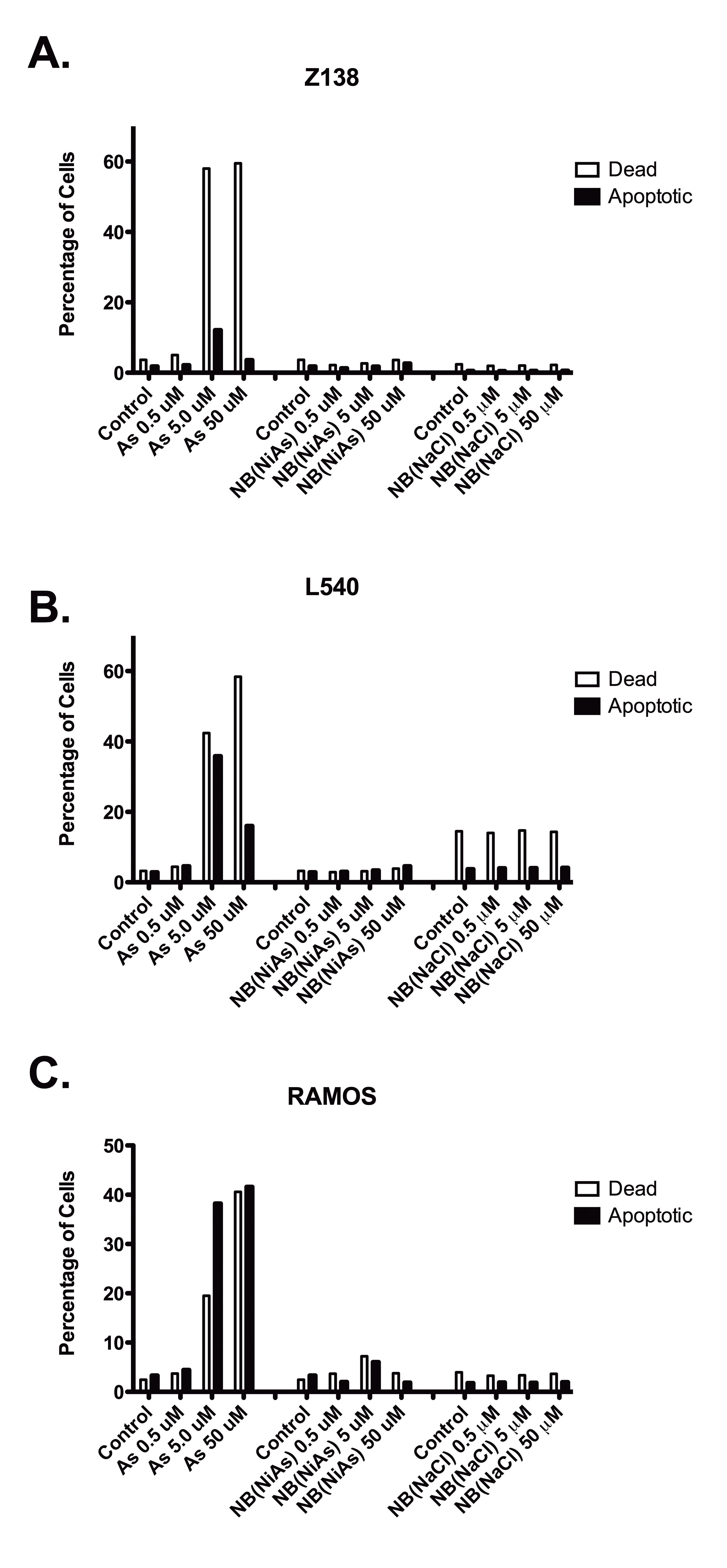

Supplement: Figure S2 — Induction of apoptosis by As2O3, NB(NaCl), or NB(Ni,As). Apoptosis was measured by staining cells with Annexin V and DAPI after treatment with 0.5, 5.0 or 50 µM As2O3, NB(NaCl), or NB(Ni,As) for 18 hr. As2O3 induces apoptosis in Z138(A), L540 (B) and RAMOS (C), while NB(Ni,As) and NB(NaCl) did not. Representative results from multiple trials. (TIFF) [file pone.0058491.s002.tif]

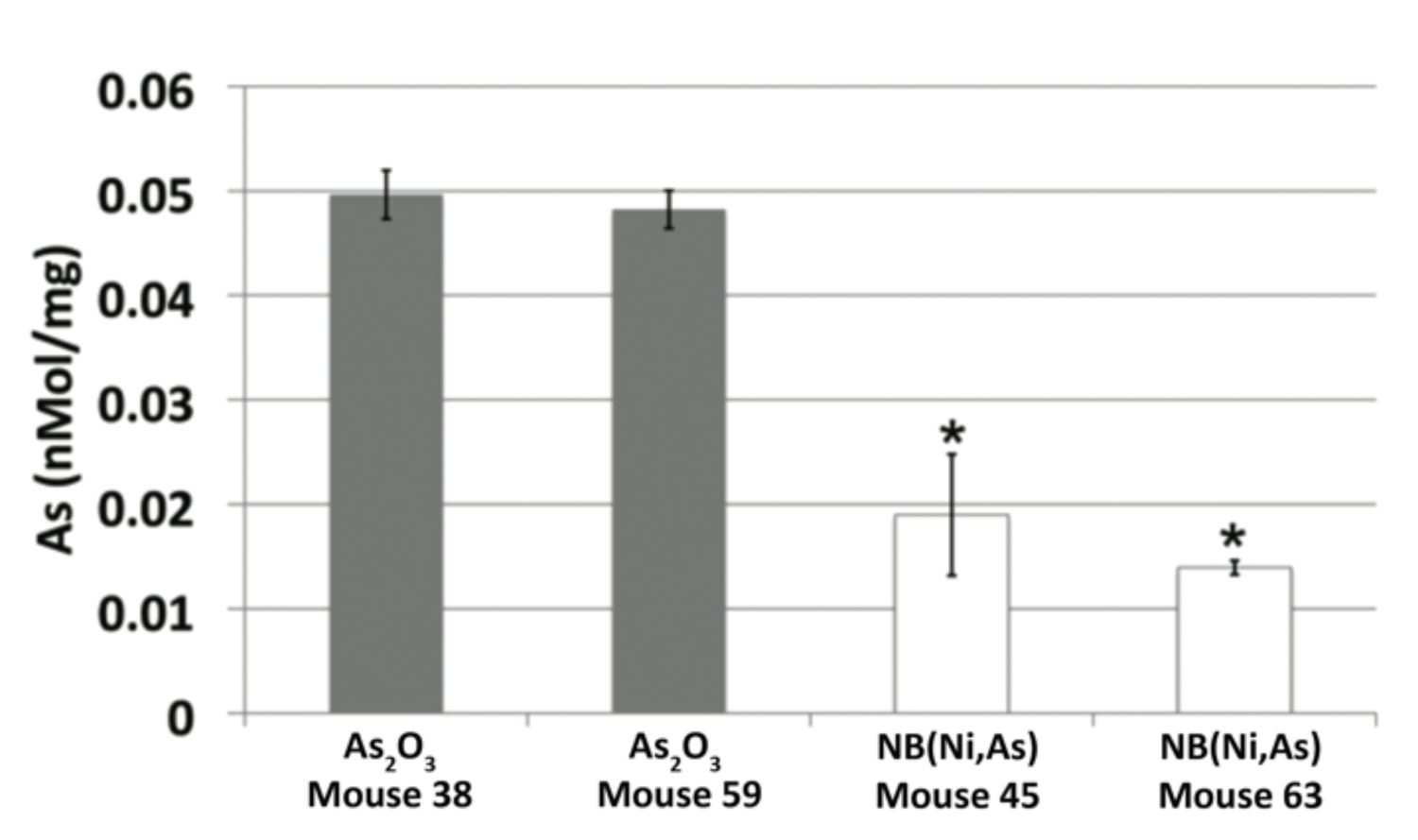

Supplement: Figure S3 — Cumulative uptake of arsenic in ovaries treated with As2O3 and NB(Ni,As). Ovaries isolated from mice treated with 4 mg/ml As2O3 or NB(Ni,As) for 3.5 weeks were analyzed for arsenic content by ICP-MS. Ovaries from mice treated with NB(Ni,As) showed significantly less arsenic uptake compared with ovaries from mice treated with As2O3. Asterisks represent P<0.01, error bars represent ± SEM. (TIF) [file pone.0058491.s003.tif]
